# A Practical Treatise on Poisons: Their Symptoms, Antidotes, and Mode of Treatment

**Published:** 1848-08

**Authors:** 


*A Practical Treatise on Poisons: their Symptoms, Antidotes, and Mode of Treatment.* By O. H Costill, M. D. 18mo., pp. 160. Grigg, Elliott & Co. Philadelphia, 1848.

In offering this Treatise to the profession, the author modestly disclaims all pretension to originality; but as the works which for the most part contain the information embodied in it are chiefly occupied with subjects of jurisprudence, they are from their size "somewhat inconvenient as books of reference for practical purposes." From the infrequency of cases of poisoning, the author observes, "the antidotal or general treatment of such cases" cannot be as familiar to the physician as the management of diseases which he is daily called to attend, "yet when such cases do occur, he is summoned in haste, and expected to act with promptness." Dr. Costill's object has been to supply "a small manual containing the symptoms and treatment of poisoning, which might be referred to in a few minutes," and thus save precious time, as well as much embarrassment to the practitioner. The object is a laudable one, and the general plan of the book is good, but we regret to find numerous evidences of hasty composition in so small a book, and on a subject in which accuracy is particularly important. The very title page is sadly marred in this way. The author, we dare say, did not mean to call it a Treatise on the *Symptoms of Poisons*, and the *Treatment of Poisons!* In the names of the poisonous articles enumerated, too, some confusion exists: thus—Turpeth Mineral is called the "Sub-Sulphate or *Peroxide* of Mercury." (page 55.) "*White Precipitate*—Ammonia, Chloride of Mercury." (p. 61.)

In speaking of poisoning with Iodide of Potassium, it is simply said, under the head of *Treatment*, "There is no antidote to this poison. It should be removed as speedily as possible by the *stomach-pump*." But suppose there is no "stomach-pump" at hand, as is very likely to be the case, is there nothing to be done?

We have likewise observed that the names of some of the authors quoted are very curiously spelled; but this is of less consequence.

We do not mention these inaccuracies and omissions to condemn the book, which is really what it professes to be—a useful manual—but to impress on the author the necessity of a careful revision in a future edition.

